# Kinase inhibitor screening identifies CDK4 as a potential therapeutic target for melanoma

**DOI:** 10.3892/ijo.2015.3097

**Published:** 2015-07-21

**Authors:** T. MAHGOUB, A.J. EUSTACE, D.M. COLLINS, N. WALSH, N. O'DONOVAN, J. CROWN

**Affiliations:** 1 National Institute for Cellular Biotechnology, Dublin City University, Dublin, Ireland; 2 Department of Medical Oncology, St. Vincent's University Hospital, Dublin, Ireland

**Keywords:** melanoma, kinase inhibitor library, cyclin dependent kinase, fascaplysin, PD0332991, CDK4

## Abstract

Despite recent advances in targeted therapies and immunotherapies metastatic melanoma remains only rarely curable. The objective of the present study was to identify novel therapeutic targets for metastatic melanoma. A library of 160 well-characterised and potent protein kinase inhibitors was screened in the BRAF mutant cell line Sk-Mel-28, and the NRAS mutant Sk-Mel-2, using proliferation assays. Of the 160 inhibitors tested, 20 achieved >50% growth inhibition in both cell lines. Six of the 20 were cyclin dependent kinase (CDK) inhibitors, including two CDK4 inhibitors. Fascaplysin, a synthetic CDK4 inhibitor, was further tested in 8 melanoma cell lines. The concentration of fascaplysin required to inhibit growth by 50% (IC_50_ value) ranged from 0.03 to 0.22 μM. Fascaplysin also inhibited clonogenic growth and induced apoptosis. Sensitivity to PD0332991, a therapeutic CDK4/6 inhibitor was also evaluated in the melanoma cell lines. PD0332991 IC_50_ values ranged from 0.13 to 2.29 μM. Similar to fascaplysin, PD0332991 inhibited clonogenic growth of melanoma cells and induced apoptosis. Higher levels of CDK4 protein correlated with lower sensitivity to PD0332991 in the cell lines. Combined treatment with PD0332991 and the BRAF inhibitor PLX4032, showed additive anti-proliferative effects in the BRAF mutant cell line Malme-3M. In summary, targeting CDK4 inhibits growth and induces apoptosis in melanoma cells *in vitro*, suggesting that CDK4 may be a rational therapeutic target for metastatic melanoma.

## Introduction

Metastatic melanoma is generally an incurable neoplasm. Few patients achieve meaningful clinical responses to conventional cytotoxic chemotherapy. In recent years, there have been significant developments in immunotherapy and molecular targeted therapy for the treatment of metastatic melanoma. Antibodies targeting immune inhibitory checkpoints, specifically CTLA4 and PD1/PD-L1 have produced meaningful improvements in melanoma survival rates (1). Inhibitors targeting BRAF produce frequent, but temporary responses in patients with V600E mutations (2). Alternative strategies to target the BRAF signalling are also been tested including MEK inhibitors, and both BRAF and MEK inhibitors are now approved as single agent therapies for metastatic melanoma. Furthermore, combining the BRAF and MEK inhibitors may further improve response rates (3).

These new therapies have resulted in improvements the 5-year survival rates for patients with metastatic melanoma but there is a need for new targets and therapies to further improve survival rates. The aim of the present study was to identify novel therapeutic targets by screening a library of 160 protein kinase inhibitors, including cyclin dependent kinase (CDK) inhibitors.

Protein kinases play central roles in the regulation of cell proliferation, differentiation and apoptosis. Deregulated kinases are often found to be oncogenic and can be central to the survival of cancer cells (4,5). Progression through the cell cycle is dependent on CDKs, a family of serine/threonine kinases that consist of a catalytic subunit known as CDK and a regulatory subunit known as a cyclin. Many of the genes involved in cell cycle progression are frequently mutated in human cancers and deregulated CDK activity can be considered a hallmark of malignancy (6–9).

The tumour suppressor p16INK4a, encoded by the CDKN2A gene, is inactivated by mutation, deletion or promoter methylation in 30–70% of melanomas (10). A 2010 study by Jonsson *et al* (11) demonstrated p16INK4a mutation, promoter methylation or lack of expression occurred in 16, 25 and 82% of melanoma metastases, respectively. The p16INK4a protein binds to CDK4/6 and inhibits interaction with D-type cyclins, which would otherwise stimulate passage through the G1 phase of the cell cycle. The frequent loss of p16INK4a in melanomas suggests that CDK4 activity may be unchecked in melanoma and may play a role in promoting uncontrolled proliferation of melanoma cells. Furthermore, mutation or overexpression of CDK4, combined with amplification of cyclin D1, has been implicated in resistance to BRAF inhibition in V600E-mutated melanoma cells, and amplification of cyclin D1 is detected in ~17% of BRAF V600E-mutated human metastatic melanomas (12).

The druggable nature of kinases has sparked considerable interest in pursuing CDKs as novel targets in anticancer drug development. Selective inhibition of CDKs may limit the progression of a tumour cell through the cell cycle and facilitate the induction of apoptosis (6,13).

## Materials and methods

### Cells and reagents

Malme-3M, Sk-Mel-2, Sk-Mel-5, Sk-Mel-28, M14 and Lox-IMVI melanoma cell lines were obtained from the Department of Developmental Therapeutics, National Cancer Institute (Bethesda, MD, USA). WM-115 and WM-266-4 melanoma cell lines were obtained from the European Association Culture Collection (UK). Malme-3M, Sk-Mel-2, Sk-Mel-5, Sk-Mel-28, M14 and Lox-IMVI cell lines were maintained at 37°C with 5% CO_2_ in RPMI-1640 medium (Sigma-Aldrich, Co. Wicklow, Ireland) with 10% fetal calf serum (FCS; Lonza, Tewkesbury, UK). WM-115 and WM-266-4 were maintained at 37°C with 5% CO_2_ in minimal essential medium (MEM; Sigma-Aldrich) with 10% FCS (BioWhittaker, Walkersville, MD, USA), 2 mM L-glutamine (Life Technologies, Dublin, Ireland), 1 mM non-essential amino acids (Life Technologies) and 1 mM sodium pyruvate (Life Technologies). Stock solutions of fascaplysin (Merck Millipore, Watford, UK) (10 mM), PLX4032 (Sequoia Research Products Ltd., Pangbourne, UK) (10 mM), elacridar (Sigma-Aldrich) (10 mM) and temozolomide (Sigma-Aldrich) (103 mM) were prepared in dimethyl sulfoxide (DMSO) PD0332991 (provided by Pfizer, Peapack, NJ, USA) (10 mM) was prepared in ultrapure water.

### InhibitorSelect™ 384-well protein kinase inhibitor library I

The InhibitorSelect protein kinase inhibitor library I (Merck Millipore) was supplied with 160 protein kinase inhibitors in a 384-well plate at a volume of 25 μl and a concentration of 10 mM in DMSO and were stored at −80°C. Stock solutions (1 mM) were prepared by dilution in DMSO, and stored at −20°C. Initial screening of the 160 protein kinase inhibitors was performed at 1 μM concentration on the Sk-Mel-2 and Sk-Mel-28 cell lines. Cells/well (1×10^3^) were seeded in 96-well plates. Plates were incubated overnight at 37°C followed by addition of drugs at the appropriate concentrations and incubated for a further 5 days until wells were 80–90% confiuent. At completion of the assay the colorimetric acid phosphatase assay was used to determine cell viability.

### Proliferation assays and acid phosphatase assay

All cells lines were seeded at 1×10^3^ cells/well in 96-well plates except for Malme-3M and WM-115 which were seeded at 2×10^3^ cells/well. Plates were incubated overnight at 37°C followed by addition of drug at the appropriate concentrations and incubated for a further 5 days until wells were 80–90% confluent. All media were removed and the wells were washed once with phosphate-buffered saline (PBS; Sigma-Aldrich). Paranitrophenol phosphate substrate (7.25 mM; Sigma-Aldrich) in 0.1 M sodium acetate buffer with 0.1% Triton-X (Sigma-Aldrich) pH 5.5 was added to each well and incubated at 37°C for 2 h. To stop the reaction 50 μl of 1 M NaOH was added and the absorbance was read at 405 nM (reference, 620 nM).

### Clonogenic assays

Malme-3M were seeded at 600 cells/well and Sk-Mel-2 were seeded at 125 cells/well in a 24-well plate. The cells were incubated overnight at 37°C. Media were removed and the drugs were added at the appropriate concentrations. Drug containing medium was removed after 4 days and replaced with drug-free media. The medium was replenished every 4/5 days thereafter. Malme-3M cells were incubated for 17 days and Sk-Mel-2 cells were incubated for 14 days. When ready to stain, media were removed and the cells washed gently with PBS twice. The cells were then fixed in cold Methacare solution (4°C, 75% v/v methanol, 25% v/v acetic acid) for 30 min. The Methacare solution was removed and the cells were washed twice with PBS before staining with 1% crystal violet (Cruinn Diagnostics, Dublin, Ireland) for 1 h. The cells were then rinsed with water and left to dry. Stained colonies were counted by eye.

### Cell cycle assays

Malme-3M cells (2.5×10^4^ per well) were seeded in 24-well plates and incubated overnight at 37°C. Fascaplysin was then added at the appropriate concentration and the plates incubated at 37°C for 48 h. Media was collected and the wells washed once with PBS. Cells were trypsinised and added to the media collected for each sample. Cells were centrifuged at 300 × g for 5 min and the media aspirated. The cell pellet was resuspended in 150 μl PBS and transferred to a round bottomed 96-well plate. The plate was centrifuged at 300 × g for 5 min and the supernatant aspirated leaving ~15 μl in each well. The remaining volume was used to resuspend the cells and 200 μl of ice cold 70% ethanol was added. The plates were then stored at −20°C for 2 h. After fixing, the cells were spun at 450 × g for 5 min, the supernatant removed, washed with 200 μl of PBS and spun again at 450 × g. The PBS was then removed and 200 μl of Guava cell cycle reagent (Merck Millipore) was added to each well. The cells were mixed by pipetting and stored at room temperature shielded from the light for 30 min. Cells were analysed on the Guava easyCyte (Merck Millipore) and the data were analysed using ModFit LT software (Verity Software House, Topsham, ME, USA).

### Terminal DNA transferase-mediated dUTP nick end labelling (TUNEL) assay

The TUNEL assays were performed using the Guava TUNEL kit for fiow cytometry (Merck Millipore), according to the manufacturer's protocol. Briefiy, 2.5×10^4^ cells were seeded per well in 24-well plates and incubated overnight at 37°C, followed by addition of drug at the appropriate concentrations. After 48 h, media were collected and the wells washed once with PBS. Cells were trypsinised and added to the media collected for each sample. Cells were centrifuged at 300 × g for 5 min and the medium was aspirated. The pellet was re-suspended in 150 μl of PBS and transferred to a round bottomed 96-well plate. A total of 50 μl of 4% paraformaldehyde (Sigma-Aldrich) made up in PBS was added to the wells and mixed. Cells were incubated at 4°C for 60 min. The plate was centrifuged at 300 × g for 5 min and the supernatant aspirated leaving ~15 μl in each well. The remaining volume was used to resuspend the cells and 200 μl of ice cold 70% ethanol (Sigma-Aldrich) was added to the cells. The plates were then stored at −20°C for 2 h. After fixation, the cells including positive and negative controls were spun at 300 × g for 5 min. Cells were washed a further 2 times at 300 × g with wash buffer. The wash buffer was aspirated and 25 μl of DNA labelling mix was added to each well and the cells mixed. The plates were covered with parafilm and incubated for 60 min at 37°C. Rinsing buffer (200 μl) was then added to each well and the plates spun at 300 × g for 5 min. The supernatant was aspirated and 50 μl of anti-BrdU staining mix added to each well, with the plate stored in the dark, at room temperature for 30 min. At the end of the incubation 150 μl of rinsing buffer was added to each well. Cells were analysed on the Guava EasyCyte (Merck Millipore). Positive and negative controls supplied with the kit were performed with each assay.

### Protein extraction and western blotting

RIPA buffer (500 μl; Sigma-Aldrich) with 1× protease inhibitors, 2 mM phenylmethanesulphonylfiouride (PMSF) and 1 mM sodium orthovanadate (Sigma-Aldrich) was added to cells and incubated on ice for 20 min. Following centrifugation at 16,000 × g for 10 min at 4°C the resulting lysate was stored at −80°C. Protein quantification was performed using the bicinchoninic acid (BCA) assay (Life Technologies). Protein (40 μg) in sample buffer [3 mM Tris HCl; 5% sodium dodecyl sulphate (SDS); 12.5% β-mercaptoethanol; 29% glycerol; 0.1% bromophenol blue] was heated to 95°C for 5 min and proteins were separated on 10% pre-cast Tris-glycine gels (Lonza). The resolved proteins were then transferred to nitrocellulose membranes (Life Technologies) using the iBlot transfer system (Life Technologies). The membrane was blocked with 5% milk powder (Bio-Rad Laboratories, Hempstead, UK) in 0.1% PBS-Tween at room temperature for 1 h, then incubated overnight at 4°C in primary antibody with 0.1% PBS-Tween in 5% milk powder. Primary antibodies used were anti-CDK4 (1:1,000; Santa Cruz Biotechnology, Heidelberg, Germany), anti-cyclin D1 (1:1,000; Cell Signaling Technology, Leiden, The Netherlands), anti-Rb (1:1,000; Cell Signaling Technology), anti-phospho-Rb (1:3,000; Cell Signaling Technology), and anti-α-tubulin (1:1,000; Sigma-Aldrich). The membrane was washed 3 times with 0.5% PBS-Tween and then incubated at room temperature with anti-mouse (Sigma-Aldrich) or anti-rabbit (Sigma-Aldrich) secondary antibody in 5% milk powder with 0.5% PBS-Tween for 1 h. The membrane was washed three times with 0.5% PBS-Tween followed by one wash with PBS alone. Detection was performed using Luminol (Santa Cruz Biotechnology) or ECL™ Advance western blotting detection kit (GE Healthcare, Buckinghamshire, UK).

### Statistical analysis

IC_50_ values and combination index (CI) values were calculated using CalcuSyn software (Biosoft, Cambridge, UK). The Student's t-test was used to determine the significance of the effects of fascaplysin and PD0332991 on the levels of cell cycle arrest and apoptosis induction, where a P-value of <0.05 was deemed significant. The Pearson's correlation was used to examine the relationship between expression of CDK4, cyclin D1, Rb and phospho-Rb by western blotting and sensitivity to fascaplysin and PD0332991.

## Results

### Melanoma cells are sensitive to cyclin dependent kinase inhibitors

Of the 160 protein kinase inhibitors tested 29 achieved ≥50% growth inhibition in the Sk-Mel-2 cell line when tested at 1 μM concentration. Twenty compounds achieved ≥50% growth inhibition in the Sk-Mel-28 cell line when tested at the same dose. The 20 compounds that achieved ≥50% growth inhibition in the Sk-Mel-28 cell line achieved similar levels of inhibition in the Sk-Mel-2 cell line (Table I). CDK inhibitors represented 6 of the 20 compounds. Four CDK1/2 inhibitors were identified, achieving between 55.3–99.8% growth inhibition in Sk-Mel-28 cells and 62.9–99.6% growth inhibition in Sk-Mel-2 cells. Two CDK4 inhibitors, CDK4 inhibitor III and fascaplysin, achieved 72.6 and 99.2% growth inhibition in Sk-Mel-28 cells and 53.9 and 98.0% growth inhibition in Sk-Mel-2 cells, respectively.

### Fascaplysin inhibits the growth of melanoma cells

**Figure 1:**
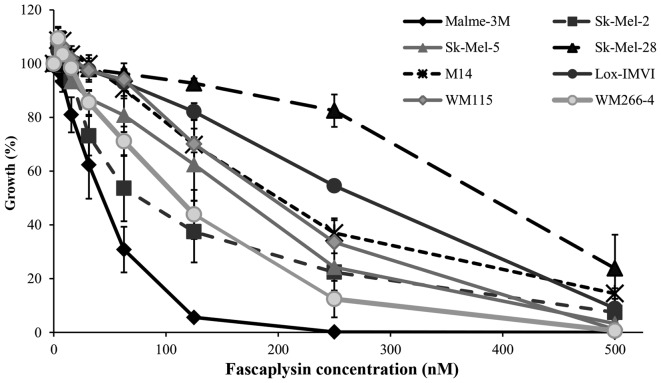
Fascpalysin in melanoma cells. Percentage growth inhibition by synthetic fascaplysin in a panel of 8 melanoma cell lines. Error bars represent the standard deviation of triplicate independent experiments.

**Figure 2:**
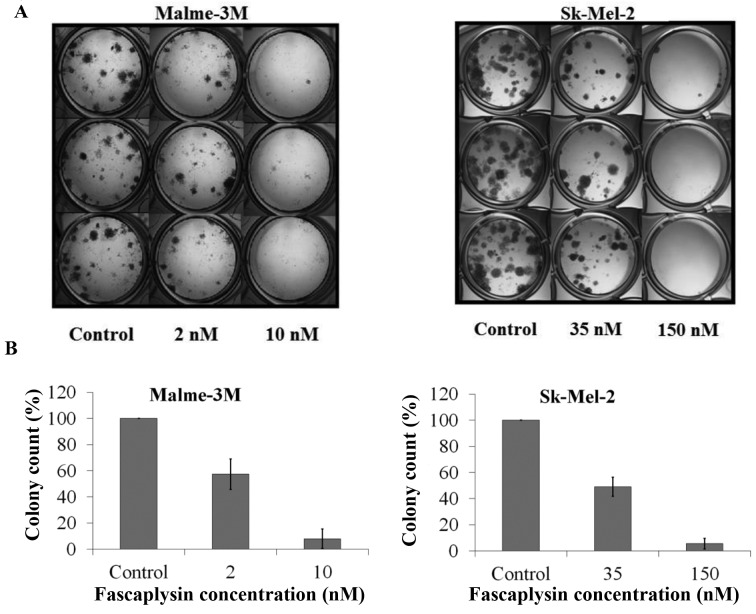
Effect of synthetic fascaplysin on clonogenic growth of melanoma cells. Representative images of clonogenic assays for (A) Malme-3M and (B) Sk-Mel-2 cells treated with fascaplysin are shown above the triplicate results. Error bars represent the standard deviations of triplicate independent experiments.

The anti-proliferative effect of synthetic fascaplysin was further investigated in a panel of 8 melanoma cell lines. All cell lines were sensitive to fascaplysin at nanomolar concentrations, with IC_50_ values ranging from 32.9 to 221.3 nM (Fig. 1 and Table II). The NRAS mutated Sk-Mel-2 cells and the most sensitive BRAF mutated cell line Malme-3M were selected for further testing. Fascaplysin significantly inhibited the clonogenic growth of both Malme-3M and Sk-Mel-2 cells (Fig. 2).

### Fascaplysin induces apoptosis in melanoma cell lines

**Figure 3:**
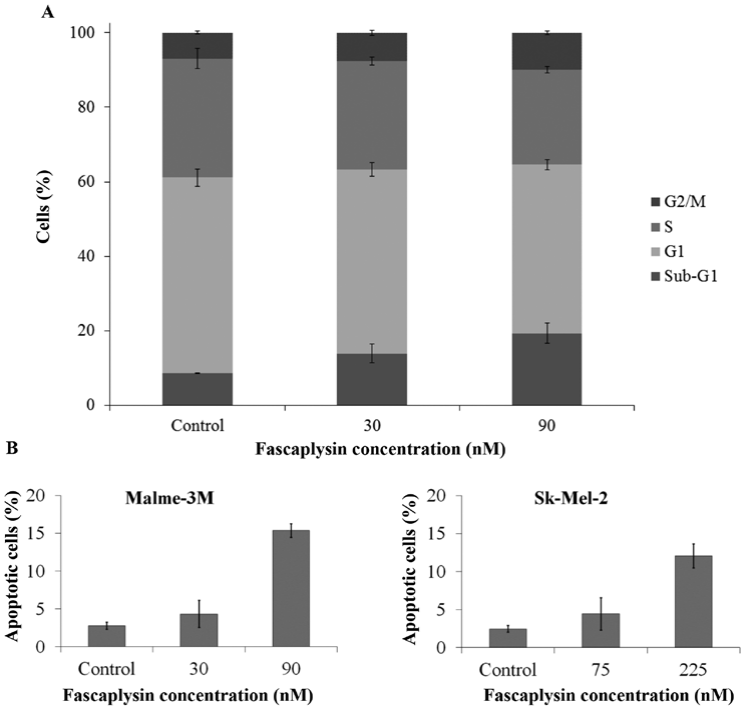
Fascaplysin induces apoptosis. (A) Cell cycle assay results for Malme-3M cells treated with fascaplysin, showing the percentage of cells in the subG1, G1, S and G2/M phases of the cell cycle. Cells were untreated (control) or treated with variable concentrations of fascaplysin. (B) The percentage apoptosis induction caused by fascaplysin treatment, measured by TUNEL assay, in Malme-3M and Sk-Mel-2 cells. Error bars represent the standard deviations of triplicate independent experiments.

To determine if the anti-proliferative effects of fascaplysin were cytostatic or cytocidal, we performed cell cycle analysis. In Malme-3M cells, 48-h treatment with fascaplysin induced a significant increase in the subG1 fraction (Fig. 3A), to 19.3±2.7% in response to 90 nM fascaplysin (P=0.020) compared with a subG1 fraction of 8.6±0.1% for control. To confirm the increase in the subG1 fraction was due to apoptosis induction, we used the TUNEL assay. Treatment with fascaplysin caused a small but significant increase in apoptosis in both Malme-3M cells (90 nM, 15.3±0.9%; P=0.0002) and Sk-Mel-2 cells (225 nM, 12.1±1.6%; P=0.006) (Fig. 3B).

### Evaluation of PD0332991 activity in melanoma cells

**Figure 4:**
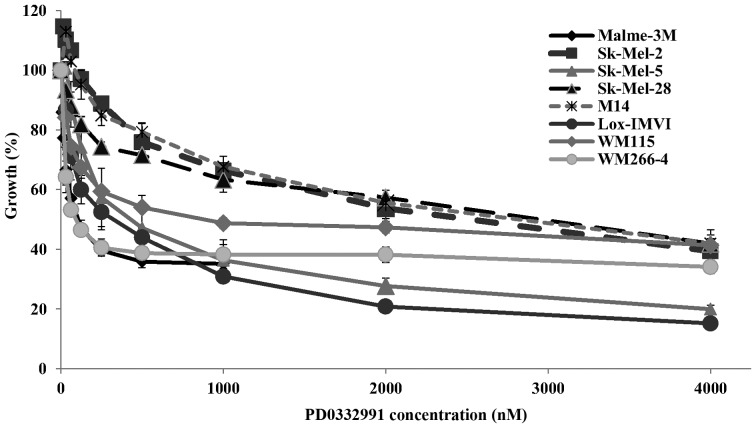
Sensitivity to the therapeutic CDK4/6 inhibitor PD0332991. Percentage growth inhibition by PD0332991 in a panel of 8 melanoma cell lines. Error bars represent the deviation of triplicate independent experiments.

As fascaplysin, a laboratory grade CDK4 inhibitor, showed significant activity in the melanoma cell lines, we tested a therapeutic CDK4/6 inhibitor PD0332991 in the panel of melanoma cell lines. Surprisingly, although PD0332991 is a more potent inhibitor of CDK4 (IC_50_=0.011 μM compared to 0.35 μM for fascaplysin), the melanoma cell lines were generally less sensitive to PD0332991 than to fascaplysin with IC_50_ values ranging from 0.13 to 2.29 μM (Table II and Fig. 4). The Sk-Mel-2, Sk-Mel-28, M14 and WM-115 cell lines showed relative resistance to PD0332991 with IC_50_ values >1 μM. Notably, the WM-266-4 cell line, derived from a metastatic tumour, was sensitive, while the WM-115 cell line, derived from the primary tumour from the same patient, was resistant to PD0332991.

**Figure 5:**
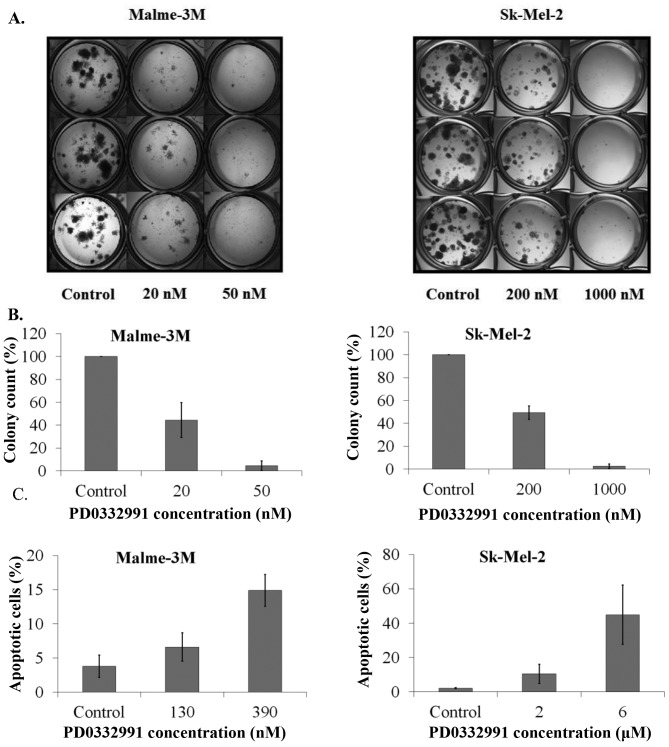
PD0332991 inhibits clonogenic growth and induces apoptosis. Effect of PD0332991 on the clonogenic growth of (A) Malme-3M and (B) Sk-Mel-2 cells. (C) Percentage apoptosis induction by PD0332991, measured by TUNEL assay, in Malme-3M and Sk-Mel-2 cells. Error bars represent the standard deviations of triplicate independent experiments.

PD0332991 also inhibited clonogenic growth and induced apoptosis in Malme-3M and Sk-Mel-2 cells, albeit at higher concentrations than fascaplysin (Fig. 5A and B). In the sensitive Malme-3M cells, 48-h treatment with PD0332991 (390 nM) induced apoptosis in 14.9±2.3% of cells (P=0.004). PD0332991 induced apoptosis in 44.9±17.2% of Sk-Mel-2 cells following 48-h treatment at a dose of 6 μM (P=0.05) (Fig. 5C).

**Figure 6:**
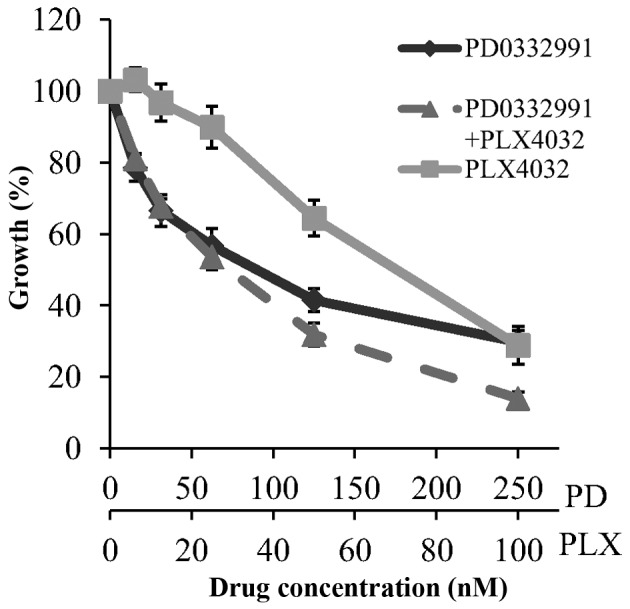
Dual targeting of CDK4 and BRAF. Effect of combining PD0332991 with PLX4032 on proliferation of Malme-3M cells, using a fixed combination ratio of 2.5:1 PD0332991 to PLX4032. Error bars represent the standard deviations of triplicate independent experiments.

Drug combination assays were performed to examine the effect of PD0332991 combined with temozolomide and the BRAF inhibitor PLX4032. Concurrent treatment with PD0332991 and temozolomide did not improve response compared to PD0332991 alone (data not shown). However, the combination of PD0332991 and PLX4032 was found to be additive, with a combination index (at ED_50_) of 1.02±0.09, in the Malme-3M cell line (Fig. 6).

### CDK4 expression correlates with response to PD0332991

To determine if the differential response to PD0332991 in the melanoma cell lines is due to drug effiux we tested the effect of an inhibitor of the drug effiux pumps P-glycoprotein and breast cancer resistance protein (BCRP), in two cell lines which displayed resistance to PD0332991, SK-Mel-2 and WM115. Elacridar (GF120918, 0.5 μM) did not enhance response to PD0332991 in either of the cell lines (data not shown).

**Figure 7:**
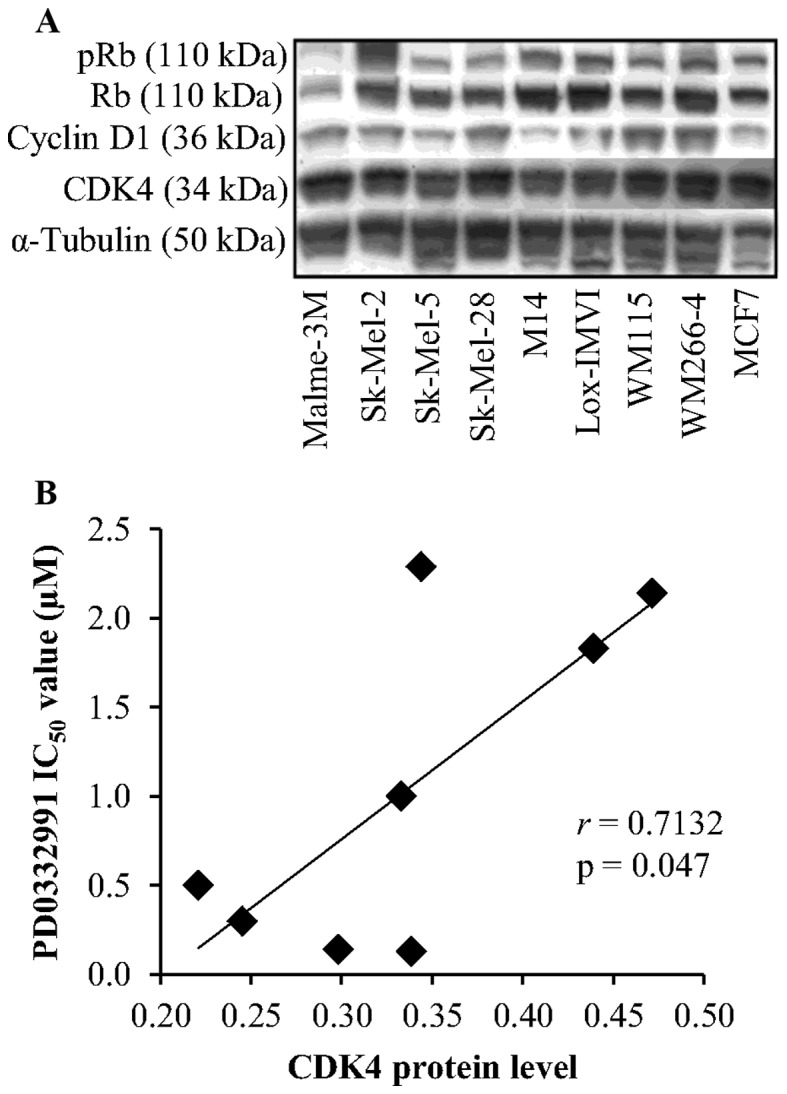
Potential PD0332991 predictive biomarkers. (A) pRb, Rb, cyclin D1 and CDK4 protein expression relative to α-tubulin determined by western blotting in a panel of 8 untreated melanoma cell lines. The MCF7 breast cancer cell line was used as a positive control. (B) Correlation between CDK4 protein levels, determined by densitometry of triplicate western blots, and sensitivity to PD0332991 in the panel of 8 melanoma cell lines.

CDK4, cyclin D1, Rb and phospho-Rb protein levels were evaluated by western blotting. CDK4 protein was detected at high levels in the panel of 8 melanoma cell lines (Fig. 7A). No correlation was observed between expression of CDK4 and sensitivity to fascaplysin (r=0.036, P=0.933). In contrast, high levels of CDK4 expression were significantly associated with reduced sensitivity to PD0332991, that is higher IC_50_ values (r=0.713, P=0.047) (Fig. 7B). No significant correlation was observed between sensitivity to either fascaplysin or PD0332991 and cyclin D1, Rb and phospho-Rb levels.

## Discussion

To identify novel targets for treatment, we screened a library of 160 protein kinase inhibitors in 2 melanoma cell lines. For a disease representative approach, we selected 2 melanoma cell lines with genetic mutations common to melanoma. The Sk-Mel-2 cell line carries an NRAS mutation, NRAS is mutated in 18% of melanomas (range, 0–50%) (14). The Sk-Mel-28 cell line is BRAF mutated, BRAF is mutated in 41% of melanomas (range 22–72%) (14). NRAS and BRAF mutations are believed to be mutually exclusive in melanoma (15).

Six of the 20 most effective compounds were CDK inhibitors. This result is not surprising considering the role of CDKs in cell cycle control. Progression through the mammalian cell cycle is a tightly regulated process. Along with their associated cyclins, CDKs are master regulators of cell proliferation. More than 11 CDKs have been identified, with CDKs 1–4 and 6 directly involved in driving the cell cycle. Deregulated CDK activity results in uncontrolled proliferation and represents a hallmark of malignancy (16).

Activating mutations, or amplification of CDK4 have been described in both familial and sporadic cases of melanoma (17). Intact p16INK4a inhibits CDK4 activity and loss of p16, which occurs frequently in melanoma, results in abnormal CDK4 activity (18). Amplification of cyclin D, which binds to CDK4, can drive cell cycle progression, and has been observed in a subset of melanomas (19). These factors provide evidence that CDK4 plays an important role in melanoma progression. Thus, we chose to further evaluate CDK4 inhibition using fascaplysin, a synthetic selective CDK4 inhibitor.

Fascaplysin was originally isolated as a compound showing antimicrobial activity from the sponge *Fascaplysinopsis* sp (20). It is a selective inhibitor of CDK4 (IC_50_=0.35 μM) and CDK6 (IC_50_=3.4 μM) and not selective for the other CDKs or other kinases (21,22). Fascaplysin, or derivatives of fascaplysin, have shown direct antitumour activity, by inducing apoptosis, and anti-angiogenic effects in preclinical tumour models, including sarcoma (23) and a number of the National Cancer Institute panel of cell lines (24).

All melanoma cell lines tested were sensitive to fascaplysin at concentrations in the nanomolar range. Notably, WM-266-4 cells were significantly more sensitive to growth inhibition by fascaplysin than WM-115 cells. WM-115 is a primary melanoma cell line, while WM-266-4 is a metastatic cell line, derived from the same patient (25). The different sensitivity may be related to the faster proliferation rate of the metastatic cell line WM-266-4 (26). The other cell lines used in this study are all derived from metastatic tumours, and all show similar sensitivity to fascaplysin as the WM-266-4 cell line, with the exception of Lox-IMVI which shows intermediate sensitivity. The lower sensitivity in the primary WM-115 cells, suggests that primary melanomas might be less sensitive to CDK4 inhibition than metastatic tumours but this would require further testing in a larger panel of cell lines derived from primary and metastatic tumours.

Consistent with previous studies showing that fascaplysin can induce apoptosis *in vitro* and *in vivo* (23,27) we showed that fascaplysin increases the subG1 fraction and apoptosis induction in melanoma cells.

Although fascaplysin has not been tested in cancer patients, several multi-target CDK inhibitors are currently being evaluated in clinical trials (5). PD0332991 is a highly specific inhibitor of CDK4 (IC_50_=0.011 μM) and CDK6 (IC_50_=0.016 μM) (28) and is currently in phase II trials in several solid tumours. One phase II study is testing PD0332991 in refractory solid tumours including stage IV melanoma (NCT01037790).

Four of the 8 melanoma cell lines tested were sensitive to PD0332991. Loss of retinoblastoma protein (Rb) has been correlated with resistance to PD0332991 (29), however, all of the melanoma cell lines tested expressed detectable levels of Rb. Analysis of the mutational status of the panel of cell lines using the COSMIC database (http://cancer.sanger.ac.uk/cancergenome/projects/cell_lines/) and the Cancer Cell Line Encyclopedia (http://www.broadinstitute.org/ccle), suggest that mutations in CDK4 or p16INK4A do not predict response or resistance, as only one of the melanoma cell lines (SK-MEL-28) has a mutation in CDK4 and there are no reported mutations in p16INK4A in these cell lines. Sanchez-Martinez *et al* (30) previously reported that PD0332991 is a substrate for the drug effiux pumps P-glycoprotein (P-gp) and breast cancer resistance protein (BCRP). Therefore, we tested elacridar, an inhibitor of P-gp and BCRP, but it had no impact on response to PD0332991 in the cell lines tested, suggesting that P-gp and BCRP do not play a role in resistance to PD0332991 in the melanoma cell lines. However, P-gp expression has been reported in ~50% of primary melanomas and 74% of metastatic melanomas (31), and thus may impact on sensitivity to PD0332991 in melanoma patients.

Using semi-quantitative western blotting, we found that lower CDK4 protein levels correlated with sensitivity to PD0332991 in the panel of 8 cell lines. A recent study showed that 37 out of 47 melanoma cell lines were sensitive to PD0332991 *in vitro* (GI_50_ <1 μM). Sensitivity to PD0332991 did not correlate with CDK4 mRNA expression in that study, but low levels of CDKN2A mRNA, or mutation or loss of CDKN2A correlated with sensitivity (32). Further evaluation of CDK4 protein levels in a larger panel of cell lines, and ultimately in tumour samples from melanoma patients treated with PD0332991, would be required to definitively determine the clinical relevance of CDK4 protein as a predictive biomarker for PD0332991 response.

The reason for the differential sensitivity to fascaplysin and PD0332991 is not fully understood. It may be due to the reported ability of fascaplysin to bind to and intercalate into DNA (33).

Although combinations of either fascaplysin or PD0332991 with temozolomide, a derivative of dacarbazine commonly used to treat melanoma, did not show enhanced anti-proliferative effects, combined treatment with PD0332991 and the therapeutic BRAF inhibitor PLX4032 was additive in the BRAF mutated cell line tested. A recent study by Jalili *et al* (34) showed that dual inhibition of CDK2 and CDK4 enhanced response to BRAF and MEK inhibitors in melanoma cells *in vitro* and *in vivo*. Thus, combining CDK4 inhibition with BRAF or MEK targeted therapies may provide greater therapeutic benefit than combinations with chemotherapy.

In summary, using kinase inhibitor screening, we showed that melanoma cells are particularly sensitive to CDK inhibition *in vitro*. Further testing of the therapeutic CDK4 inhibitor PD0332991 showed that 4 out of 8 melanoma cell lines tested are sensitive to CDK4 inhibition and that CDK4 inhibition induces apoptosis in melanoma cells. Our results suggest that CDK4 inhibition represents a promising approach for the treatment of metastatic melanoma.

## Figures and Tables

**Table I:** Percentage growth inhibition (± standard deviation) by the 20 most active compounds tested at 1 μM in Sk-Mel-2 and Sk-Mel-28 melanoma cell lines.

	% Growth inhibition	
	
	Sk-Mel-28	Sk-Mel-2
Akt inhibitor IV	86.3±6.4	95.3±1.7
Alsterpaullone, 2-Cyanotheyl	65.6±13.0	64.5±4.1
Aurora kinase/CDK inhibitor	55.3±21.8	62.9±5.6
CDK 1/2 inhibitor III	99.9±0.0	98.0±1.0
CDK 4 inhibitor III	72.6±22.8	53.9±8.5
Cdk/crk inhibitor	99.8±0.0	99.6±0.2
EGFR inhibitor	87.7±5.7	94.3±0.3
Fascaplysin, synthetic	99.2±0.6	98.0±1.0
Herbimycon A, streptomyces sp.	73.9±15.3	89.6±3.8
IC261	87.5±7.0	93.8±1.2
JNK inhibitor	91.5±1.3	95.9±1.0
K-252a, Nocardiopsis sp.	93.3±0.6	92.8±3.3
MK2a inhibitor	92.6±1.9	93.9±1.9
PDGF receptor tyrosine kinase inhibitor IV	89.9±1.0	95.3±1.4
PDK1/AKT/Flt dual pathway inhibitor	99.9±0.0	99.8±0.3
PKR inhibitor	68.0±2.4	83.7±3.3
PI-103	89.0±1.3	79.2±6.7
Rapamycin	66.8±5.0	65.5±5.9
Staurosporine, streptomyces sp.	99.2±0.3	99.6±0.4
TGF-β RI inhibitor III	75.5±3.5	74.5±8.2

**Table II:** IC_50_ concentrations of fascaplysin (nM) and PD0332991 (μM) in a panel of melanoma cell lines.

	Mutation status	Fascaplysin (nM)	PD0332991 (μM)
Sk-Mel-2	NRAS	74.5±6.1	2.14±0.24
Malme-3M	BRAF	32.9±1.7	0.14±0.01
Sk-Mel-5	BRAF	93.0±11.1	0.50±0.05
Sk-Mel-28	BRAF	220.0±19.1	1.83±0.18
M14	BRAF	178.7±23.4	2.29±0.28
Lox-IMVI	BRAF	221.3±18.4	0.30±0.03
WM115	BRAF	166.6±25.9	1.00±0.11
WM266-4	BRAF	87.5±13.5	0.13±0.01

Standard deviations represent the deviation of triplicate independent experiments.
